# Eye Movements Index Implicit Memory Expression in Fear Conditioning

**DOI:** 10.1371/journal.pone.0141949

**Published:** 2015-11-12

**Authors:** Lauren S. Hopkins, Douglas H. Schultz, Deborah E. Hannula, Fred J. Helmstetter

**Affiliations:** Department of Psychology, University of Wisconsin-Milwaukee, Milwaukee, WI, 53211, United States of America; University of Lethbridge, CANADA

## Abstract

The role of contingency awareness in simple associative learning experiments with human participants is currently debated. Since prior work suggests that eye movements can index mnemonic processes that occur without awareness, we used eye tracking to better understand the role of awareness in learning aversive Pavlovian conditioning. A complex real-world scene containing four embedded household items was presented to participants while skin conductance, eye movements, and pupil size were recorded. One item embedded in the scene served as the conditional stimulus (CS). One exemplar of that item (e.g. a white pot) was paired with shock 100 percent of the time (CS+) while a second exemplar (e.g. a gray pot) was never paired with shock (CS-). The remaining items were paired with shock on half of the trials. Participants rated their expectation of receiving a shock during each trial, and these expectancy ratings were used to identify when (i.e. on what trial) each participant became aware of the programmed contingencies. Disproportionate viewing of the CS was found both before and after explicit contingency awareness, and patterns of viewing distinguished the CS+ from the CS-. These observations are consistent with “dual process” models of fear conditioning, as they indicate that learning can be expressed in patterns of viewing prior to explicit contingency awareness.

## Introduction

Pavlovian fear conditioning has been particularly useful in studies designed to identify brain structures and systems involved in different aspects of learning and memory. Differential Pavlovian fear conditioning is a procedure in which a neutral conditional stimulus (CS+) is presented together with an aversive unconditional stimulus (UCS). With repeated pairings, the CS+ comes to evoke a conditional response (CR). In contrast, an alternate stimulus (CS-), never paired with the UCS, can serve as a control, and does not elicit a CR [1]. Exposure to the conditioning procedure promotes associative learning, which can be indexed in a variety of ways. Typically, evidence for learning is obtained using measures of physiological arousal (e.g. skin conductance response (SCR), heart rate changes) and/or explicit reports (e.g., a post-experimental questionnaire (PEQ) designed to assess conscious contingency knowledge).

Whether or not explicit knowledge (i.e. awareness) is required for successful fear conditioning is currently a matter of debate. While some investigators have proposed that a single propositional learning mechanism drives conditioning [2, 3], others have argued that conditioning can occur with or without concomitant awareness of learned associations (i.e., dual process theory) [4–7]. According to a “strong” version of the single process model, the ability to explicitly articulate stimulus relationships is an obligatory prerequisite for expression of a conditioned response whereas a “weak” version of the model postulates that the learning process gives rise to both contingency awareness and CRs in tandem [2, 8]. These models are similar in that they both propose a close correspondence between contingency awareness and CRs. In other words, differences in physiological responses that have traditionally been described as *implicit* indices of learning (e.g. a more robust SCR to the CS+ than the CS-) should not occur in the absence of declarative knowledge for the CS-UCS relationship. Consistent with this proposal, the results of several experiments indicate that successful differential conditioning is only evident among participants who can explicitly identify the CS+ and the CS- [9–13].

Others have proposed that implicit and explicit learning can develop independently during training (i.e. “dual process” theories of conditioning). According to this view, associative learning can occur in the absence of explicit contingency knowledge [4, 14], which is typically assessed using PEQ responses. Furthermore, in the event that a Pavlovian conditioning procedure results in both differential implicit and explicit responses, it does so via the parallel activation of separate memory systems rather than as a result of a single propositional learning process. Some of the best evidence in favor of this perspective comes from neuropsychological investigations that implicate the amygdala in autonomic CR expression and the hippocampus in declarative memory for learned contingencies [15–17].

While dissociations reported in neuropsychological investigations provide strong support for the dual process theory and for the independence of brain systems that give rise to different expressions of learning, evidence in favor of the view that conditioning can occur absent awareness in neurologically intact individuals is required as well. Notably, recent investigations suggest that CR expression does not depend on explicit knowledge when healthy college-age participants are tested [5, 18–20]. In one of these studies perceptual similarity of the CS+ to the CS- was systematically manipulated [5]. For one group, the CS+ and the CS- were easily discriminable, and for another group they were not. Critically, when discriminability was sufficiently difficult, participants did not develop explicit contingency knowledge. However, both groups had larger magnitude SCRs to the CS+ than to the CS-, an outcome that suggests awareness is not necessary for CR expression. Other experiments using different methods (e.g. subliminal stimulus presentation) [21] or alternative dependent measures of learning (e.g., startle and eye blink) [22–24] have also supported these conclusions.

Despite emerging experimental support for the dual process theory, results have frequently been questioned on methodological grounds [3, 25, 26]. A key criticism concerns the standard procedures used to evaluate awareness. Contingency awareness has most often been assessed via PEQs which are administered after the test session is complete. However, the use of PEQs as a valid measure of awareness has been challenged [2, 27]. For example, it has been argued that participants may forget the contingencies in the time period between completion of the experiment and PEQ administration, especially if that period is long, the experimental task is difficult, or other events have been attended. In other words, participants may have been aware of the imposed contingencies during task administration, but classified as unaware based on PEQ responses. In addition, because they are administered at the end of the experiment, PEQs are a temporally insensitive measure of awareness. Participants are typically categorized as aware of the contingencies or not, and no information about *when* awareness developed is available. Consequently, investigators cannot evaluate the conditioned response (e.g., as indexed by SCR, startle, or eye blink) *before and after* the onset of awareness in order to determine whether and how results vary as learning accrues.

More recently, investigators have started to incorporate online measures of awareness (e.g., trial-by-trial key presses or ratings) that permit participants to report whether or not materials presented on a given trial are likely to be paired with the UCS [18, 28–30]. This method is potentially quite sensitive, and is especially powerful if the temporal specificity afforded by the approach is leveraged when analyses are performed. Simply classifying participants as having achieved awareness or not based on evaluation of the concatenated data (e.g., [27, 28]) is useful, but more can be done. Here, we take full advantage of the temporal specificity afforded by use of an online measure and evaluate data separately for pre- and post-aware trials taking into account the fact that rates of learning can vary across individual participants.

Another potential concern about results reported in past fear conditioning studies has to do with frequent use of SCR as the sole index of learning. The problem with this approach is illustrated by investigations that have shown dissociations across measures (i.e. startle and SCR), with SCR only sensitive to learning among aware participants [22]. More generally, it has been argued that standard indices of the conditioned response, like SCR, may be insufficiently reliable as they are subject to habituation and individual differences in responsivity [2]. Inconsistencies in the reliability of SCR as an index of conditioning are particularly problematic when this measure is used in isolation to support the hypothesis that awareness is either necessary or not for the production of CRs [5, 9, 20, 21]. Consequently, the identification of additional, more sensitive measures would be useful.

One method that has been used successfully to index memory in the absence of awareness entails recording and evaluation of eye movements to visual materials [31]. Several investigations have shown that patterns of viewing distinguish studied from novel materials [32–34], and that eye movements are sensitive to memory for studied inter-item or item-context relationships [35–38]. Especially important in the context of the current study, a subset of these experiments have shown that memory-based differences in patterns of viewing can be documented even when explicit memory is not diagnostic of past experience [36, 39]. For example, participants look disproportionately at regions of scenes that have been manipulated despite an inability to explicitly describe the change [36, 39, 40], and eye movements distinguish studied from novel items even when participants incorrectly endorse novel items as studied [34]. This is not to say that eye movements are insensitive to, or unaffected by conscious appreciation of encoded content, but rather that they can serve as a reliable index of learning and memory with and without awareness (cf. [41]). Consistent with these outcomes, eye movements may prove useful in the evaluation of associative learning during conditioning.

In addition to eye-movement-based indices of learning, a second measure that may be of use in investigations of fear conditioning is pupil size. Past work has demonstrated that anticipation of an aversive event is associated with greater pupil dilation during the presentation of a conditioned stimulus [42–44]. Furthermore, by concurrently measuring SCR and heart rate (measures of sympathetic and parasympathetic nervous system activity, respectively) during presentation of affective visual stimuli, one study found that pupil size covaried with SCR but not with cardiac output [45]. It was concluded that both pupil size and SCR reflect sympathetic nervous system activity and are sensitive to emotional arousal in the presence of affective stimuli. Given the discrepant findings associated with the use of SCR in awareness studies, combined assessment of SCR and pupil size, as is done here, will permit comparative evaluation of these measures, and will provide some insight into whether or not pupil size is a more sensitive and consistent measure of arousal than SCR. This is important because, as noted above, pupil size and SCR are thought to be controlled by similar autonomic mechanisms.

The goal of the current experiment was to combine several measures of learning (i.e. SCR, eye movements, and pupillometry) with real time assessment of contingency knowledge to address questions about whether or not awareness is required for the expression of a conditional response. Rather than use simple stimuli (e.g. colored squares) as has typically been done in the past [5, 46], we developed an approach using a complex visual scene that was presented repeatedly over the course of the experiment. Across trials, different items were embedded in the scene. One of these items (present in half of the trials) was always paired with aversive electrical stimulation (i.e., CS+); a second item (present in the remaining trials) was never paired with the aversive event (i.e., CS-). Participants provided explicit expectancy ratings (i.e., a conscious index of whether or not they expected to receive a shock) on every trial and SCRs, eye movements, and pupil size were recorded. The use of a continuous online measure of contingency knowledge meant that we could compare pre- and post-awareness periods during training, while use of a standard PEQ would permit assessment of explicit knowledge upon completion of the experiment. Furthermore, evaluation of several measures sensitive to learning (i.e. SCR, eye movement, and pupil size) allowed for a more comprehensive characterization of the conditional response than is typical in the literature (although see [22] for a similar approach with SCR and fear-potentiated startle). Eye movement recording in particular affords us the opportunity to identify a new, sensitive index of associative learning that may yield additional evidence in favor of the dual-process perspective.

Based on past work with standard recognition memory tasks [37], we predicted that participants would look disproportionately at the CS+ and the CS- relative to matched comparison objects embedded in the same scenes. Moreover, it was predicted that this effect would be evident in advance of explicit contingency knowledge. It was also predicted that more time would be spent viewing the CS+ than the CS-. While both of these items have predictive value, previous findings suggest that *intrinsically* aversive stimuli (e.g. spiders) are more likely to capture and hold attention (and viewing) than neutral stimuli [47–49]. Whether or not similar effects occur in the context of fear conditioning tasks when a neutral stimulus acquires aversive properties via learning has not yet been examined. Finally, it was expected that pupil size and SCR amplitude would be greater for CS+ than CS- trials and that these effects would be seen prior to the development of explicit contingency knowledge. These outcomes would be consistent with past investigations indicating that learning develops quickly in fear conditioning studies and can occur in the absence of explicit contingency knowledge [5, 21, 43].

## Materials and Methods

### Participants

Twenty-four right-handed undergraduates participated in this study and were compensated with extra credit. Four participants were dropped from reported analyses because eye movements could not be reliably recorded. Therefore, reported results are based on a sample of 20 participants (15 women; mean age = 20.25, *SEM* = .5). All procedures were approved by the Institutional Review Board at the University of Wisconsin—Milwaukee, and written informed consent was obtained from each participant before the experiment began.

### Apparatus

#### Expectancy Dial

Participants manipulated a custom made rotary dial attached to their right thigh with a Velcro strap to report UCS expectancy (see [50] for a video example of the appearance and use of the dial). The center of the dial included an elongated notch which could be rotated 90 degrees to the left and right. Expectancy data was sampled at a rate of 40 Hz.

#### Electrical Stimulus and Skin Conductance (SCR) Recordings

The UCS was a 500ms electrical stimulus delivered via an AC (60Hz) source (Contact Precision Instruments, Model SHK1) through two surface cup electrodes (silver/silver chloride, 8 mm diameter, Biopac model EL258RT). These electrodes were placed over the right tibial nerve above the medial malleolus as in prior work [5]. A Contact Precision Instruments unit with a SC5 24-bit digital amplifier was used to record skin conductance at a rate of 1000 Hz. Skin conductance data were collected with electrodes (BIOPAC Model EL258RT) that were filled with electrolytic gel and attached to the sole of the left foot 2 cm apart. PSYLAB software was used for skin conductance data analysis.

#### Eye Tracking and Stimulus Delivery System

An Applied Science Laboratories D6 remote eye tracker was used to record eye position and pupil size. Eye position coordinates were acquired approximately every 17ms (i.e. at a 60-Hz rate). Presentation software (Neurobehavioral Systems) was used to control stimulus delivery, and the visual images were presented on a Dell Optiplex 980 desktop computer equipped with an Asus 19-inch LCD monitor.

### Materials & Design

**Fig 1:**
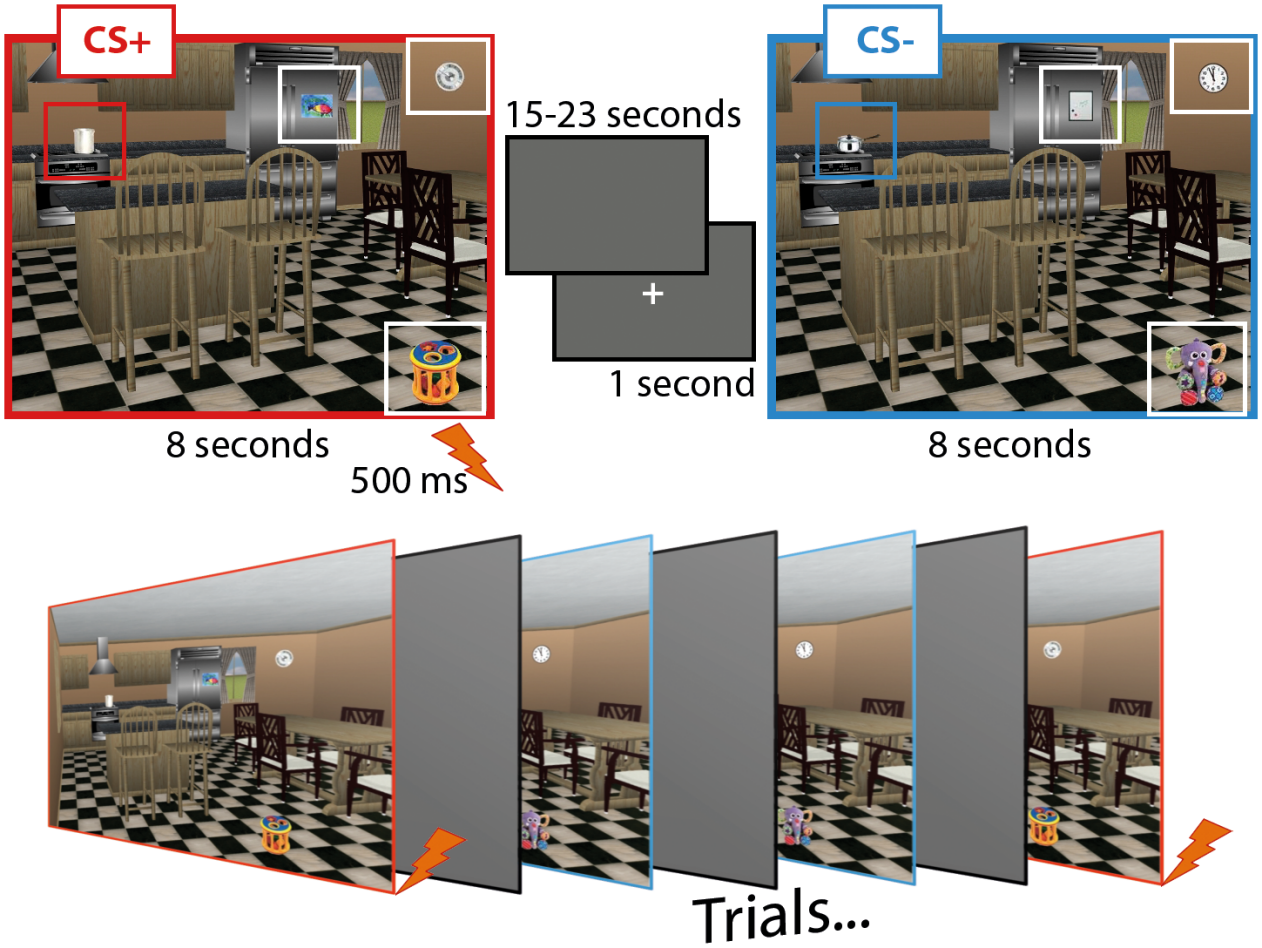
Materials & Trial Structure. Materials that were used in this experiment are shown here along with specific information about event timing. Colored borders, and boxes that surround the objects embedded in each scene are for illustration only. In this example, scenes with red borders contain the CS+ (i.e. a tall pot) and co-terminate with the UCS; those with blue borders contain the CS- (i.e. a sauce pot) and do not co-terminate with the UCS. Three additional objects (i.e. a magnet, a clock, and a toy), each with two exemplars that were presented interchangeably across trials, are enclosed in white boxes. These objects were fillers and each exemplar was presented with the UCS on 50% of the trials.

Materials for this experiment were slightly different exemplars of the same rendered kitchen scene. Each scene was developed with Punch! Home Design software (Encore Software Inc., Eden Prairie, MN), and was sized to 800 by 600 pixels. Four items (i.e., a pot, a clock, a refrigerator magnet, and a toy) were embedded in each scene, and these items remained in the same spatial locations across scene exemplars. Two different versions of each item were selected from the web using Google Image and any background content was removed with GIMP software. One item from each pair was embedded in the kitchen scene on every trial, creating a complex visual stimulus (Fig 1). The purpose of creating stimuli more complex than those commonly used in Pavlovian fear conditioning experiments (e.g. colored shapes on a black background) was to reduce rapid CS+ identification so that trials could be binned into pre- and post-awareness periods. All of the possible item permutations were used, resulting in a total of 16 unique kitchen scene exemplars; across exemplars, individual items from each pair were presented eight times.

From the four embedded items, two were selected to act as CSs for counterbalancing purposes—i.e., the pot and the toy. For half of the participants, the pot was the CS. In this case, one pot (from the pair) was the CS+ while the remaining pot was the CS-. Each item from the pair was used as a CS+ and a CS- equally often across participants. For the remaining participants, the toy was the CS, and the same counterbalancing procedures were used. The remaining items (i.e. two exemplars each of the magnet and the clock) were presented randomly and did not have any predictive value (i.e. they co-terminated with the UCS on 50 percent of the trials). These items were included to make explicit CS identification more difficult.

### Procedure

After informed consent was obtained, a UCS intensity work-up was completed and participants were instructed in the use of the UCS expectancy dial. During the work-up procedure, the maximum level of tolerable shock intensity was identified for each participant. This work-up procedure consisted of no more than five presentations of the electrical stimulus, each rated by the participant on a scale from 0 to 10 (0 = no sensation, 10 = painful but tolerable). Stimulation intensity was increased incrementally until the participant provided a rating of 10 at which point the work-up procedure was terminated and the experiment commenced. UCS intensity was subsequently maintained at the level that had been rated with a 10 during the work-up procedure (*M* = 1.7 mA, *SEM* = .27).

Following the work-up procedure, eye-position was calibrated using a 3 x 3 spatial array, and instructions were provided. Participants were told that they would see several pictures and that their eye movements would be recorded. In addition, they were told that a shock would be delivered occasionally throughout the course of the study and that they should make predictions about when this would occur. Specifically, participants were instructed to provide online ratings of shock expectancy, and were told that they should update these responses to accurately reflect their expectations throughout the experiment. When they were certain that a shock would *not* be delivered, participants were told to turn the dial all the way to the left, a response corresponding to a rating of zero. In contrast, when they were certain that a shock would be delivered, they were told to turn the dial all the way to the right, corresponding to a rating of 100. The notch of the dial was to be kept in the middle position if participants were unsure about UCS delivery. It was emphasized that the dial could be placed anywhere along this continuum (i.e. from 0 to 100), but in practice, participants rarely reported intermediate levels of awareness. At the end of a trial, participants returned the dial to the center position. No information was provided about potential relationships between the UCS and the visual materials and no visual feedback about the position of the dial was provided during the experiment, as this would affect eye movement behavior. Importantly, participants who completed this experiment were not encouraged to look for cues or given any instructions that would encourage specific search patterns when pictures were presented. Instead, we simply took advantage of the natural tendency viewers have to explore elements in scenes when they are presented, a free-viewing manipulation. Once the experimenter was satisfied that participants understood the instructions, and any remaining questions had been answered, the experiment began. The experiment consisted of 32 trials, and across trials each kitchen scene exemplar was presented twice. The CS+ (e.g. pot 1) was present in the scene for half of the trials, and the CS- (e.g. pot 2) was present in the scene for the remaining trials. Trial order was dictated by one of two possible pseudorandomized lists created with the constraint that there were no more than two consecutive trials of the same type. On every trial, the scene remained in view for 8 seconds, and when the scene contained the CS+ the trial always co-terminated with a 500ms presentation of the UCS. A variable duration intertrial interval (mean = 20 seconds) was imposed to permit rectification of the SCR and consisted of a blank gray screen (RGB = 104). Prior to scene onset, a white crosshair was presented for 1 second in the center of the screen and participants were instructed to fixate this location (Fig 1). While the trial was in progress participants were instructed to make a prediction as to the likelihood of receiving the UCS and could update their rating whenever their prediction changed as described in detail above.

Following the experiment proper, participants completed a short PEQ developed to assess explicit contingency knowledge. They were asked to indicate whether or not they felt any of the items in the scene predicted shock administration and then attempted to identify the CS+ and the CS- from forced-choice displays. Each of these displays contained all 8 of the items that had been embedded in the kitchen scene during testing. On the first presentation, participants were instructed to circle the image that predicted shock (i.e. the CS+), and when the set was re-presented, they were instructed to circle the image that predicted the absence of shock. Debriefing was provided at the end of the session.

### Data Analysis

Data analysis proceeded in two stages. First, we evaluated learning on a trial by trial basis over the course of the entire experiment. In this case, comparisons were made between progressively yoked CS+ and CS- trials (e.g. trial 1 CS+, trial 1 CS-, … trial 16 CS+, trial 16 CS-) to ensure that learning had occurred.

Next, an awareness-locked analysis was performed. UCS expectancy data were used to identify *the* trial representing the onset of awareness for each participant. UCS expectancy was defined as the mean expectancy rating recorded during the final 4 seconds of scene presentation on each trial. Based on these values, the onset of contingency awareness was defined as the *first trial* in a run of five consecutive trials where CS+ trials were rated at 75 or above and CS- trials were rated at 25 or below. Due to the pseudorandomized trial order, the five-trial sliding window included at least two presentations of the CS+ and the CS-. Participants who never met the five trial minimum were defined as having remained unaware of the imposed contingencies at the conclusion of the study (N = 5) [5], and were dropped from the awareness-locked analysis. Once the trial representing the onset of awareness had been identified for all of the remaining participants, SCR, pupil size, and eye-movement data were binned into pre- and post-aware periods. All of the trials prior to the onset of awareness were defined as *pre-awareness trials* whereas all trials subsequent to this point were defined as *post-awareness trials*. Because the onset of awareness (as defined above) varied considerably across participants (Table 1), data analysis was limited to the subset of pre- and post-aware trials immediately preceding or following the first “aware” trial. This approach ensured that the number of pre- and post-awareness trials carried forward for subsequent analyses was equated for each participant, and that we were evaluating the subset of trials just prior to, and immediately following, the development of explicit contingency knowledge. Altogether, three CS+ and three CS- trials immediately preceding the first “aware” trial defined the pre-awareness period; similarly, three CS+ and three CS- trials occurring immediately after the first “aware” trial defined the post-awareness period. Pre- and post-aware SCR, pupil, and eye movement data were then evaluated for evidence of a conditioned response.

**Table 1:** Trial Representing Awareness Onset for Participants Included in Awareness Analysis.

Participant	Overall Awareness Onset Trial	CS+ Awareness Onset Trial	CS- Awareness Onset Trial
1	10	6	5
2	15	7	9
3	23	12	12
4	16	9	8
5	7	4	4
6	11	6	6
7	7	4	4
8	16	8	9
9	7	4	4
10	16	8	9
11	13	6	8

Awareness was defined by successful differentiation of the CS+ (i.e. ratings at or above 75) from the CS- (i.e. ratings at or below 25) on five consecutive trials. The number representing the first trial, from the set of five, in which participants demonstrated awareness of the imposed contingencies from the entire set of 32 trials (overall awareness onset trial) is indicated for each participant along with the first awareness trial from the 16 CS+ and CS- trials, respectively (CS+ and CS- awareness onset trials). Data here illustrate individual differences in the progression of learning.

#### Skin Conductance

SCRs from a 2 second baseline period prior to CS+ and CS- scene onset, and from the corresponding 8 second scene presentation period, were carried forward for analysis. For all of the reported contrasts, mean baseline skin conductance level was subtracted from the peak SCR during the 8 second period when scenes were in view [51]. This was done for each trial individually. Data from individual trials were then binned by condition (CS+ or CS-) to evaluate learning.

#### Pupil Dilation

Baseline measures of pupil size were obtained while participants viewed a gray screen (RGB = 104) for a period of 1 second prior to scene onset. Pupil size was also recorded over the course of the entire 8 second CS+ or CS- scene presentation period. Blinks and other instances of loss in the raw pupil data were flagged and discarded, and individual samples that were more than three standard deviations from the mean on a given trial were discarded as outliers. Six percent of the trials (40 trials altogether; individual participant range = 0 to 3) were flagged as bad because the number of valid samples was less than 65 percent of the total possible. These trials were dropped from the awareness-locked analysis, and were replaced with values that were derived from linear interpolation of the data from neighboring trials for the trial by trial analysis [44].

As has been done previously [52], maximum pupil size during the presentation of each scene was identified for each trial individually after blinks and outliers were removed. A single peak pupil response was obtained for each trial by averaging three samples prior and subsequent to the identified peak. The peak pupil response was then evaluated in terms of percent change from the average pupil size obtained during the corresponding pre-stimulus baseline period. Individual trials were then binned into conditions of interest (CS+, CS-) for use in the reported contrasts.

#### Eye-Movement Measures

Fixations were calculated offline using the Eyenal software package (Applied Science Laboratories, Bedford, MA) with the constraint that subsequent samples be averaged together into a single fixation if changes in gaze point across samples were less than 1 degree of visual angle and had a minimum duration of 100ms.

Individual trials were removed from reported analyses when eye position was lost or unreliable. As has been done in past work [34], trials were eliminated if total viewing time to the scene was less than 65% of the 8000ms display time (i.e., less than 5200ms). Any participants with fewer than 8 trials remaining in one of the conditions of interest following this procedure were dropped from further analyses, as reported above. As was the case for pupil size data, individual trials that had been flagged as bad (based on the above criterion) were replaced with the linear trend from adjacent trials for the trial by trial analysis; bad trials were eliminated from the awareness-locked analysis.

For data analysis, the CS (e.g., the pot) was designated the *target item* while the other potential CS (e.g., the toy) was designated the *non-target comparison item*. This approach is consistent with previously published eye tracking investigations [36, 37]. All of the reported comparisons were based on the proportion of total viewing time directed to these two regions of interest (ROIs)–the location occupied by the CS (target) and the location occupied by the comparison object (non-target) (cf. Fig 1). As for the above measures, direct comparisons were also made between the CS+ and the CS-.

## Results

### Trial by Trial Analyses

To evaluate the progression of awareness and learning, three separate repeated measures ANOVAs with the factors trial type (CS+, CS-) and trial number (1–16) were calculated using UCS expectancy, SCR, and pupil size data, respectively.

**Fig 2:**
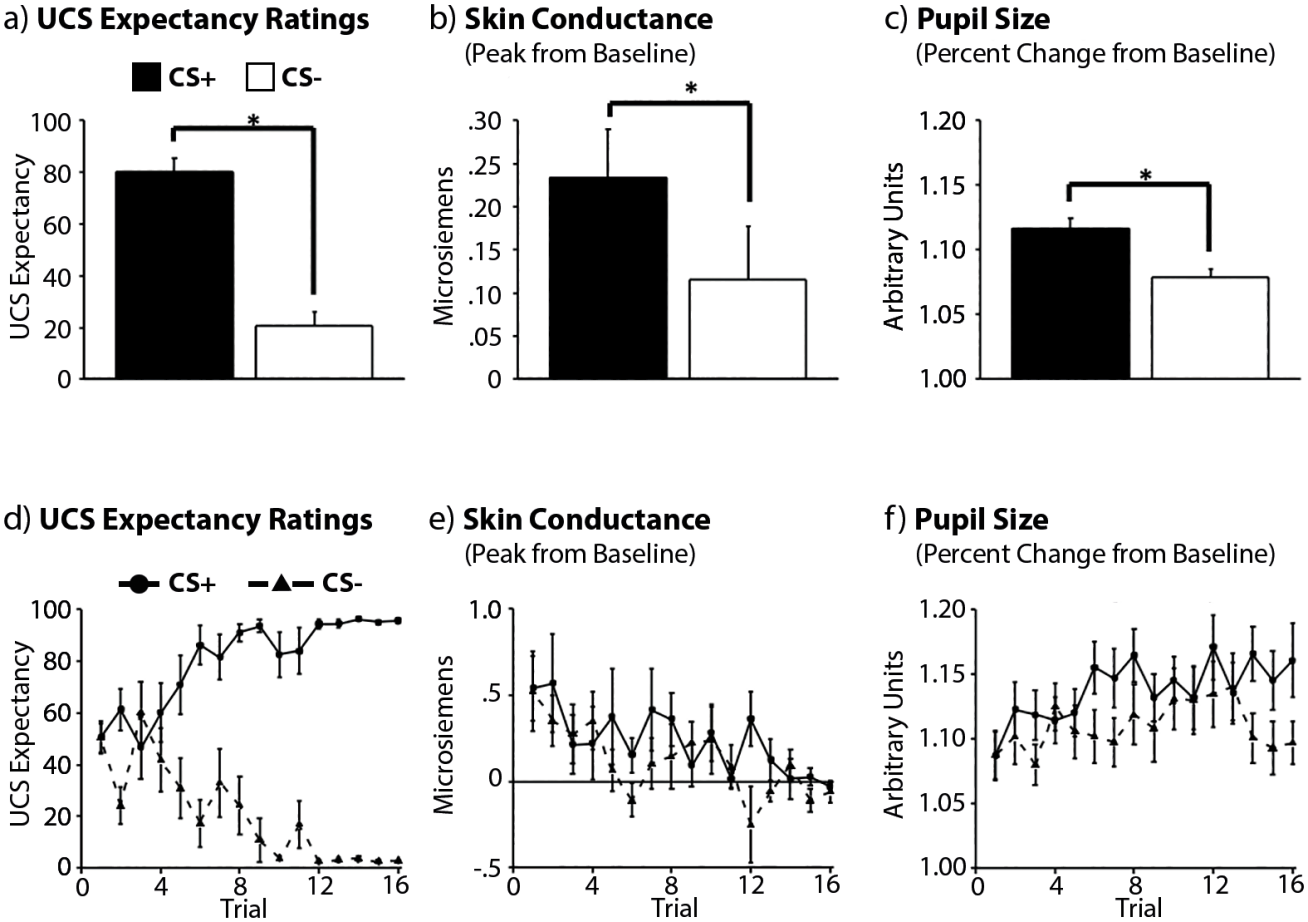
Expectancy Ratings, SCR, and Pupil Size Data. (top) Global averages, collapsed across all of the experimental trials. Participants rated the CS+ higher than the CS- in online dial-based expectancy ratings (a) and also showed larger magnitude SCRs (b) and pupil size (c) to CS+ than CS- trials. (bottom) Trial-by-trial illustration of the expectancy, SCR, and pupil dilation data. Online expectancy ratings (d) were increasingly sensitive to differentiation of the CS+ from the CS- across trials. The magnitude of SCRs (e) declined across trials, whereas pupil size (f) increased; neither SCRs nor pupil size measures showed changes in sensitivity to learning as the experiment progressed. Error bars show SEM.

Results calculated based on UCS expectancy indicated that participants could successfully distinguish CS+ from CS- trials (main effect of trial type: *F(1,19) = 48*.*17*, *p <* .*001*, *η*_*p*_^*2*^ = .*71*; Fig 2a), and that this explicit contingency knowledge emerged gradually over the course of the experiment (trial type x trial number interaction: *F(15,285) = 7*.*93*, *p <* .*001*, *η*_*p*_^*2*^ = .*3*; see Fig 2d). The main effect of trial number was not statistically reliable (*F(15*, *285)* = .*84*, *p* = .*64*, *η*_*p*_^*2*^ = .*04*). Bonferroni corrected post-hoc comparisons indicated that, on average, differences in UCS expectancy to CS+ and CS- trials were reliable ten trials into the experiment (i.e. by trial 5 for the CS+ and CS-, respectively; *t(19) = 3*.*65*, *p <* .*05*, *Cohen’s d* = .*82*). The delayed onset of awareness confirms that our difficulty manipulation was effective.

Differences in SCR magnitude and pupil size also distinguished CS+ from CS- trials (main effect of trial type: *F’s(1,19) ≥ 7*.*79*, *p’s ≤* .*05*, *η*_*p*_^*2*^ = .*29 and* .*43*, *respectively*), results that indicate both measures were sensitive to learned contingencies. The magnitude of the SCR response decreased over the course of the experiment (main effect of trial number: *F(15,285) = 5*.*83*, *p <* .*001*, *η*_*p*_^*2*^ = .*24*), an outcome that is commonly reported in the literature [53, 54], but there was no evidence for a change in the magnitude of learning across trials (trial type x trial number interaction: *F(15*, *285) = 1*.*66*, *p* = .*16*, *η*_*p*_^*2*^ = .*08*). In contrast to SCR, pupil size increased across trials (*F(15*, *285) = 1*.*7*, *p <* .*05*, *η*_*p*_^*2*^ = .*08*), but again, there was no evidence for a change in the magnitude of learning-based pupil size differences over the course of the experiment (*F(15*, *285) = 1*.*2*, *p* = .*28*, *η*_*p*_^*2*^ = .*06*). In sum, both SCR and pupil size indexed learning (see Fig 2b and 2c), but neither measure was sensitive to changes in learning as the experiment progressed (Fig 2e and 2f).

**Fig 3:**
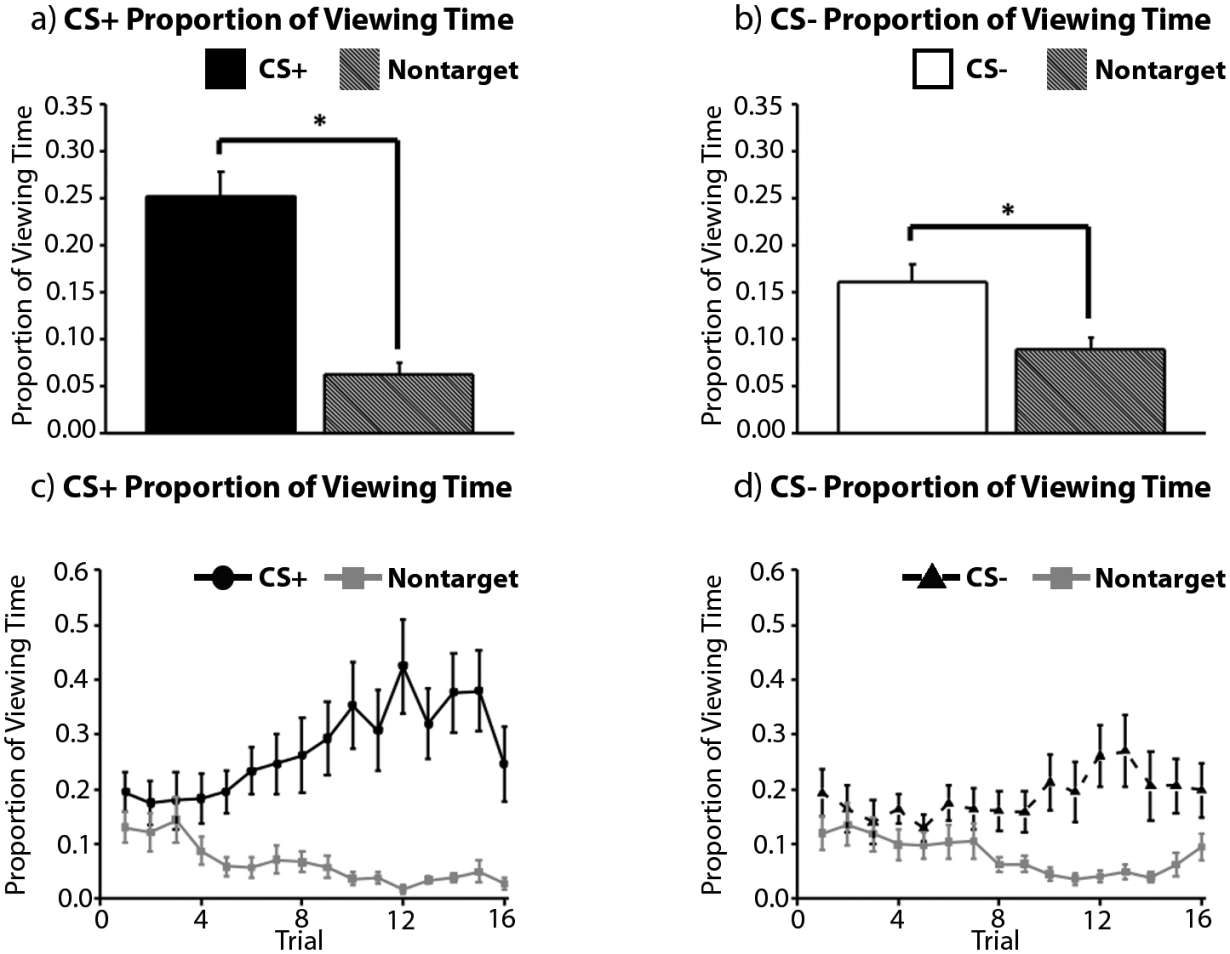
Eye Movement Data. (top) Global averages, collapsed across all of the experimental trials. Participants directed more viewing time to both the CS+ (a) and the CS- (b) than to non-target comparison objects embedded in the same scenes; this preferential viewing effect was more robust for the CS+ than the CS-. (bottom) Trial-by-trial illustration of the eye movement data. Disproportionate viewing of the CS+ (c) and the CS- (d) relative to the non-target comparison object became increasingly robust across trials, and was greater for the CS+ than for the CS-. Error bars show SEM.

A final repeated measures ANOVA with the factors trial type (CS+, CS-), item type (target, non-target), and trial number (1–16) was calculated to evaluate whether or not there was evidence for learning in patterns of eye movement behavior. As predicted, participants spent more time viewing targets (i.e. the CS+ and the CS-) than non-target items embedded in each scene (main effect of item type: *F(1*, *19) = 15*.*64*, *p <* .*001*, *η*_*p*_^*2*^ = .*45*; Fig 3a and 3b), an eye-movement-based learning effect that was more robust for pictures containing the CS+ than the CS- (trial type by item type interaction: *F(1*, *19) = 12*.*9*, *p <* .*005*, *η*_*p*_^*2*^ = .*4*). The main effect of trial number was not statistically reliable (*F(15*, *285)* = .*93*, *p >* .*05*, *η*_*p*_^*2*^ = .*05*), but the magnitude of the eye-movement-based learning effect grew as the experiment progressed (item type by trial number interaction: *F(15*, *285) = 5*.*56*, *p <* .*001*, *η*_*p*_^*2*^ = .*23*; Fig 3c and 3d, bottom). Bonferroni corrected post-hoc comparisons indicated that preferential viewing was directed to the CS+ by trial 6 (*t(19) = 3*.*59*, *p <* .*05*, *Cohen’s d* = .*84*) and to the CS- by trial 12 (*t(19) = 4*.*3*, *p <* .*001*, *Cohen’s d = 1*.*13*). These results confirm that eye movements can be used to index learning in the context of Pavlovian fear conditioning tasks, and indicate that the magnitude of this eye-movement-based learning effect is greatest when the CS+ is present in the picture, as predicted.

### Awareness-Locked Analysis

Based on the criteria for awareness outlined above, 15 participants successfully distinguished CS+ from CS- trials at some point after the start of the experiment, suggesting that they were aware of the imposed contingencies. Four of these participants learned the contingencies by trial 6, and were excluded from awareness-locked analyses because they had fewer than three pre-aware trials per condition. Therefore, data from 11 participants were carried forward for the awareness analysis. Among these individuals, onset of awareness varied from trial 7 to trial 23 (*M = 12*.*8*, *SEM = 1*.*5*; see Table 1).

**Fig 4:**
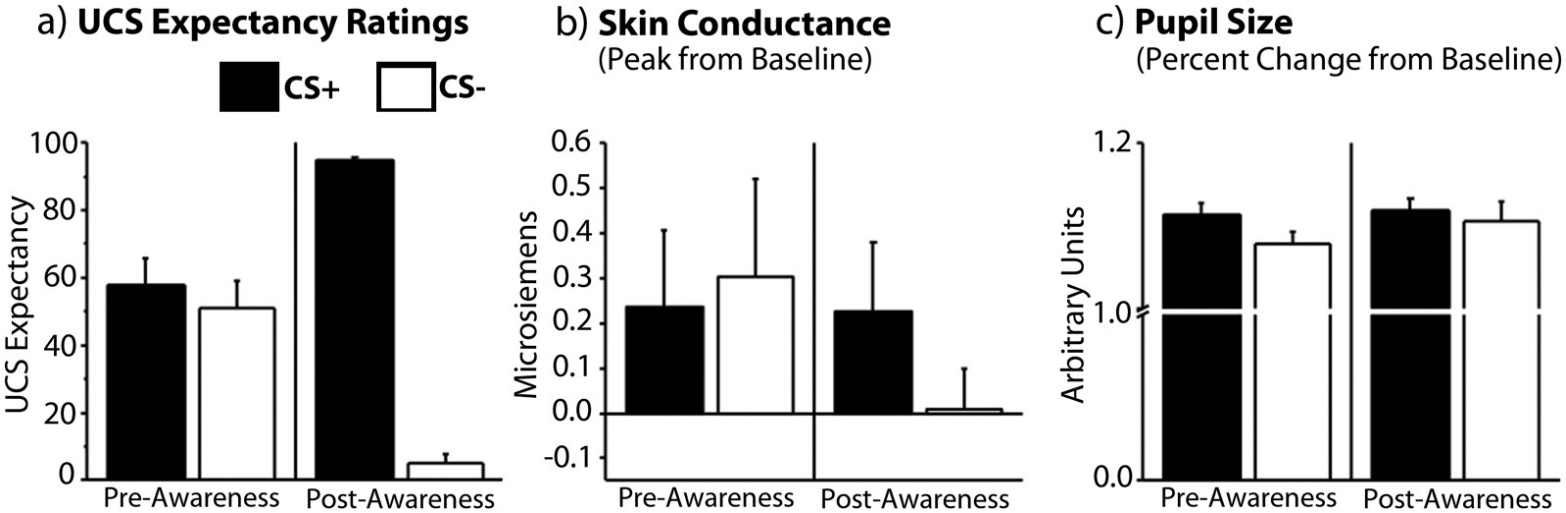
Awareness-Locked Expectancy Ratings, SCR, and Pupil Size Data. (a) Participants rated the CS+ higher than the CS- after, but not before the onset of explicit contingency knowledge as defined by a 5-trial sliding window. (b) Participants also showed higher SCR for CS+ than CS- trials post-awareness but not pre-awareness. (c) There were no significant differences in pupil size pre- or post-awareness. Error bars show SEM. N = 11.

To confirm that explicit knowledge of contingencies was only present for post-aware trials, a repeated measures ANOVA with the factors awareness (pre-aware trials, post-aware trials) and trial type (CS+, CS-) was calculated using the UCS expectancy data. Results indicated that UCS expectancy ratings were higher for CS+ than for CS- trials (*F(1*, *10) = 83*.*74*, *p <* .*001*, *η*_*p*_^*2*^ = .*89*), but this main effect was qualified by a reliable trial type by awareness interaction (*F(1*, *10) = 122*.*79*, *p <* .*001*, *η*_*p*_^*2*^ = .*93*). Direct comparisons indicated that participants could not reliably distinguish CS+ from CS- trials before the onset of explicit contingency knowledge (*t(10)* = .*67*, *p >* .*05*, *Cohen's d* = .*2*), but were successful thereafter (*t(10) = 38*.*39*, *p <* .*001*, *Cohen's d = 10*.*9*; Fig 4a). This outcome was critical, and permits us to evaluate whether or not there is any evidence for learning (in implicit measures—i.e. SCR, pupil size, and eye movements) prior to the onset of explicit contingency knowledge.

Repeated measures ANOVAs with the factors awareness (pre-aware trials, post-aware trials) and trial type (CS+, CS-) were calculated using SCR and pupil size data. Skin conductance responses were greater for CS+ than for CS- trials (*F(1*, *10) = 5*.*34*, *p <* .*05*, *η*_*p*_^*2*^ = .*35*), and there was a marginal awareness by trial type interaction (*F(1*, *10) = 4*.*07*, *p* = .*071*, *η*_*p*_^*2*^ = .*29*). As can be seen in Fig 4b, the small, statistically unreliable SCR difference prior to awareness was not in the expected direction (*t(10)* = .*368*, *p* = .*89*, *Cohen’s d* = .*11*), but the predicted effects were evident once participants had explicit knowledge of the imposed contingencies (*t(10) = 2*.*56*, *p <* .*05*, *Cohen's d = 1*.*31*). No statistically reliable differences in pupil size were identified (all *F’s ≤ 1*.*34*, *p’s >* .*05*; Fig 4c).

**Fig 5:**
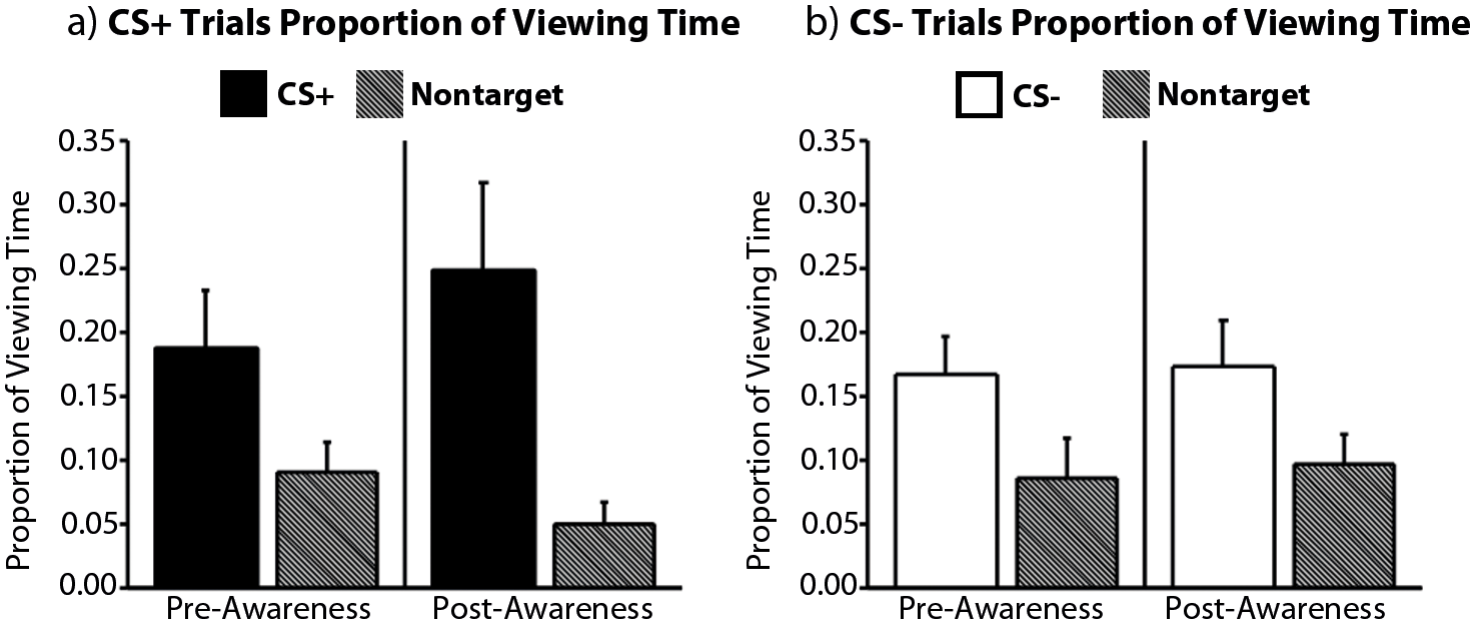
Awareness-Locked Eye Movement Data. More viewing time was directed to the CS+ (left) and the CS- (right) than to non-target comparison objects in advance of, and subsequent to, the onset of explicit contingency knowledge as indexed by dial ratings. This effect was marginally more robust in the post-aware period for scenes that contained the CS+. Error bars show SEM. N = 11.

Whether or not viewing time measures reflect learning prior to awareness was evaluated using a repeated measures ANOVA with the factors awareness (pre-aware trials, post-aware trials), trial type (CS+, CS-), and item type (target, non-target). Results confirmed that participants spent more time viewing targets (i.e. the CS+ and the CS-) than non-targets (main effect of item type: *F(1*, *10) = 11*.*18*, *p <* .*005*, *η*_*p*_^*2*^ = .*53*), and there was a trend in the expected direction for greater disproportionate viewing when the CS+ was present in the scene (*F(1,10) = 3*.*34*, *p* = .*098*). Planned comparisons indicated that the magnitude of this eye-movement-based learning effect (i.e. calculated by subtracting proportion of viewing time directed to non-targets from proportion of viewing time directed to targets, separately for CS+ and CS- trials pre- and post-awareness) was comparable for scenes containing the CS+ and the CS- before the onset of awareness (*t(10) = 1*.*03*, *p* = .*32*), but there was a trend towards more robust CS+ viewing subsequent to awareness (*t(10) = 2*.*17*, *p* = .*05*). There were no other statistically reliable main effects or interactions (all *F’s(1*, *10) ≤* .*71*, *p’s ≥*.*42*; Fig 5). These results indicate that viewing time measures can index learning in advance of explicit associative knowledge, and that this learning effect is marginally more robust for scenes that contain the CS+, particularly in the post-aware period.

### Post-Event Questionnaire Responses

PEQ responses were used to determine whether or not participants had successfully identified the link between item identity and shock administration. In response to the initial question, 15 participants indicated that shock delivery was tied to the presence of a specific item embedded in the scene.

Five participants from the original sample (n = 20) were classified as “unaware” at the conclusion of the study based on their expectancy ratings; consistent with that classification, all five of these individuals failed to successfully identify the CS+ and the CS- from among the 8 alternatives when forced-choice probe trials were administered. The remaining participants (N = 15) had been classified as “aware” based on expectancy ratings. Despite successful online discrimination of CS+ and CS- trials, four of these individuals failed to identify the CS+ and/or the CS- when the 8-alternative forced-choice probe trials were administered. However, due to their ability to successfully discriminate between the CS+ and CS- on the online measure of contingency knowledge, they were included in the awareness-locked analysis.

## Discussion

This study had two objectives. The first was to determine whether or not evidence for fear conditioning could be obtained in advance of explicit contingency knowledge. The second was to evaluate potential sensitivity of eye movements to acquisition of a conditioned fear response. Consistent with past reports [21, 55], SCRs distinguished CS+ from CS- trials, although in the current study this effect was not significant prior to the onset of awareness. The same general pattern of results was evident when pupil size was used to index learning, but reported differences between CS+ and CS- trials were not statistically reliable when analyses were limited to a smaller subset of the data (i.e. when awareness-locked comparisons were performed). Evaluation of viewing time data provided converging evidence for learning, and proved to be an especially sensitive measure. Consistent with our predictions, patterns of viewing directed to items embedded in each kitchen scene indexed the learned association between a target object and shock administration, an effect that was greater for CS+ than for CS- trials. Critically, and in contrast to other indirect measures used here, disproportionate viewing of the item that might be paired with shock (e.g. the pot) was evident before participants had explicit knowledge of the imposed contingencies. These results support the utility of eye movements in the difficult task of measuring unconscious associative learning, and do so under conditions of natural viewing using complex stimuli that are better matched to real-world experiences. Importantly, they also highlight the sensitivity of this measure to implicit learning in a conditioning paradigm when other measures were insufficient.

Previous investigations evaluating the role of awareness in fear conditioning have relied primarily on PEQ responses to index explicit contingency knowledge [13, 17, 55]. However, limitations of this approach (e.g., lack of temporal specificity, dichotomous classification of participants as aware or not) make it clear that a more sensitive assessment tool is necessary. Here, we used an online measure of awareness that not only permits differentiation of aware from unaware participants, but is also sensitive to *when* (i.e. on what trial) awareness develops. This meant that we did not have to treat individual participants as either strictly aware or unaware, and that comparisons of within-participant data from pre- and post-aware periods could be performed.

Our criterion for awareness, as indexed by the online expectancy measure, was based on a run of five consecutive dial responses that were diagnostic of picture type (i.e. CS+ vs. CS-). Data were subdivided into pre- and post-aware periods relative to the first trial in this run, an approach that takes into account differences in the time-course of learning across individual participants. That this method is especially sensitive to learning was evident in reported outcomes, as a small subset of individuals who would have been classified as unaware based on PEQ responses alone, successfully distinguished CS+ from CS- trials in online ratings. The lack of perfect correspondence between measures is not unexpected and is consistent with criticisms of the PEQ as a lone indicator of awareness, but might also mean that our online index of awareness was overly conservative reflecting the combined influence of unconscious and conscious processes on decision making [56]. If this is the case, then evidence for learning in pre-aware eye movement data is even more compelling because the first trial in our sequence of five may not correspond to fully developed explicit knowledge of the imposed contingencies.

Together with previous reports that have used SCR, fear-potentiated startle, and eye blink to index CRs [4, 5, 17, 21, 57], results from this study provide converging evidence in favor of the view that the expressions of learning in fear conditioning tasks do not require awareness. In the current study, pre-aware evidence for learning was only found in eye movement behavior; SCR and pupil size measures did not distinguish CS+ from CS- trials in advance of explicit contingency knowledge. Because pupil size was an exploratory measure, and the experiment was not optimized for this purpose (e.g., luminance differences within and across displays were not strictly controlled), the absence of statistically reliable differences in pupil size across trial types prior to awareness should not be considered conclusive. Future studies could evaluate effects of learning on pupil size more effectively by carefully controlling the perceptual characteristics of visual displays that affect this measure.

Our SCR results are similar to those reported by Weike and colleagues who found evidence for conditioning without awareness using potentiated startle, but not SCR [22, 24, 58]. There are at least two potential explanations for this pattern of results. First, it may be the case that SCR is correlated with declarative knowledge of the imposed contingencies, and that past reports of unaware SCR differences reflect insufficient assessment of awareness [3]. A second potential explanation concerns the materials and testing procedures that have been used to evaluate learning. There were several notable methodological differences between our experiment and those that have shown differential SCR without awareness in the past. For example, unlike Öhman and Soares [59], the current experiment did not use phylogenetically fear-relevant stimuli. It has been proposed [60, 61] that one of the central features of the fear system is its “selectivity,” meaning that evolutionarily threatening stimuli (e.g. snakes) will more easily activate the system than contemporary stimuli that may still be dangerous (e.g. guns). Based on this proposal, stimuli used in the Öhman and Soares study would elicit automatic activation of the fear system and result in the differential activation of unaware autonomic fear responses (e.g. SCR and startle); other aversive materials, like the ones used here (e.g. pots), would not. As a final note, it is important to point out that our materials (i.e. complex scenes), which did not lend themselves to trivial CS+/CS- discrimination, were qualitatively different from the standard in fear conditioning studies (e.g. simple colored shapes) [5, 21]. Therefore, the complexity of our materials and other task characteristics that likely required a comparatively large contribution of cognitive resources may have attenuated differential SCRs [62, 63].

Despite the potential issues associated with these methodological differences, the use of complex scenes in the current study meant not only that we could delay the onset of contingency awareness, but also that patterns of eye movements could be used as an additional, potentially sensitive, index of learning. Specifically, this approach meant that we could examine whether or not viewing (and attention) were directed preferentially to *the* item that predicted presence/absence of shock relative to a control object embedded in the same scene. One advantage of this method is that it provides us with more detailed information than SCR or pupil size measures, which themselves provide no objective information about whether or not the CS itself is being prioritized for extended processing. As predicted, viewing time measures distinguished target objects (CS+ and CS-) from non-target comparisons objects, the identities of which were counterbalanced across participants. This eye-movement-based learning effect was statistically reliable prior to the onset of awareness, and more robust when the CS+ was present in the scene. This means that attention was allocated preferentially to objects in scenes that signaled potential onset of an aversive event, and that processing was temporally extended for the target object (e.g., a specific pot) that predicted an aversive outcome. The sensitivity of eye movements to learning compliments previous reports from the memory literature that have documented changes in patterns of viewing that reflect past experience even when explicit recognition responses are inconclusive or incorrect [31–34], and go a step further by demonstrating that eye movement behavior can be used to index learning in the context of Pavlovian fear conditioning tasks. To our knowledge, this is the first demonstration that eye movements can be used as an additional measure of learning in fear conditioning investigations.

Related to the idea that eye movements can provide important information about the processing of fear-related stimuli, we have already seen in this study that differential viewing patterns are associated with items paired with aversive outcomes versus those that are not. Although disproportionate viewing of targets prior to the onset of contingency awareness was seen for both CS+ and CS- trials, characterization of viewing behavior as trials progressed revealed distinct patterns of viewing for each CS trial type. Specifically, whereas on CS+ trials participants seemed to steadily increase the amount of time they spent viewing the target, on CS- trials, the increase was less robust. These differences in viewing patterns may represent a difference in the perceptual bias towards the CS+ versus the CS-. Studies measuring EEG recordings of visual evoked potentials (VEPs) have found increased VEP amplitudes to a compound stimulus that included a CS+ but no such increase during the presentation of a compound stimulus that included a CS- [64]. The current results compliment these findings and suggest that learned cues that signal the absence of an aversive event do not obtain the same level of salience as cues that do reliably signal aversive outcomes. In the current study, pre-awareness viewing measures suggest that targets are more salient than the comparison distractor items but the CS+ and CS- are equally salient. However, as the experiment progresses and contingency awareness develops, it is likely that viewing patterns change to reflect disproportionate salience commonly associated with threat versus safety signals [64], thus reflecting additional learning that has just occurred.

Despite the differences in viewing patterns to the CS+ and the CS- post-awareness, the key finding was that preferential processing of both the CS+ and CS- was present prior to awareness. Evaluation of the brain structures and systems that support early prioritization and subsequent awareness would be an interesting avenue for future research. Consistent with previous reports [19, 43, 65], it seems reasonable to predict that activity differences in the amygdala would be sensitive to the imposed contingencies pre- and post-awareness. Exactly when and how the hippocampus would contribute is a bit more difficult to predict. While nobody would debate a role for the hippocampus in explicit learning and memory [66], recent work suggests that the hippocampus may also support encoding and retrieval of memories that are not accessible to awareness [67]. Currently, hippocampal activity differences have only been reported in conditioning studies among participants who were aware of the experimental contingencies at the end of the study [19, 43, 65], but these experiments have not evaluated patterns of hippocampal activity as contingency awareness develops (e.g. pre- and post-awareness as was done here). Therefore, it remains to be determined whether hippocampal activity is strictly tied to conscious awareness or whether activity differences may be correlated with pre-aware indices of learning (e.g., eye movement behavior).

Finally, it is important to consider limitations of the current investigation that might be addressed in future work. For example, although our parsing of data into pre- and post-awareness periods allowed us to take individual differences into account and to more precisely evaluate participant behavior at the within-subjects level, our online measure may have been insufficiently sensitive to partial awareness of the imposed contingencies. For example, participants may have learned that the pots were related somehow to shock without knowing how exactly that relationship was defined (e.g. which exemplar was the CS+). In this case, pre-aware eye movement behavior may have been correlated with some degree of contingency knowledge. On the other hand, and as indicated above, it is also possible that even successful online discrimination, as indexed by dial ratings here, reflects mixed contributions of unconscious and conscious processes; this possibility is suggested by the lack of correspondence between dial ratings and PEQ responses among a small subset of our participants. Future studies could address this issue by using a more liberal measure of awareness (e.g. by asking participants to identify the object they suspect is related to shock administration, without requiring differentiation of the CS+ from the CS).

In conclusion, the current study investigated the role of contingency awareness in classical conditioning using several implicit measures of learning (i.e., SCR, pupil size, and eye movements) and an online measure of awareness. Results support the dual-process theory of conditioning which states that explicit and implicit learning processes can occur independent of one another and that CRs can be produced in the absence of awareness. Future studies should use fMRI in tandem with eye measures to investigate the neural substrates associated with pre- and post-awareness stimulus processing. Such results could be useful in elucidating the neural components involved in the development of phobias or other disorders associated with implicit fear responses, such as post-traumatic stress disorder.

## Supporting Information

S1 Data UCS Expectancy, SCR, Pupil Size, and Eye-Tracking Data Associated with CS+ and CS- Trials. (XLSX)
